# Team climate, intention to leave and turnover among hospital employees: Prospective cohort study

**DOI:** 10.1186/1472-6963-7-170

**Published:** 2007-10-23

**Authors:** Mika Kivimäki, Anna Vanhala, Jaana Pentti, Hannakaisa Länsisalmi, Marianna Virtanen, Marko Elovainio, Jussi Vahtera

**Affiliations:** 1 Department of Epidemiology and Public Health, University College London, London, UK; 2 Department of Work Organizations, Finnish Institute of Occupational Health, Helsinki, Finland; 3 Division of Health Services Research, National Research and Development Center for Welfare and Health, Helsinki, Finland; 4 Department of Psychology, University of Helsinki, Finland

## Abstract

**Background:**

In hospitals, the costs of employee turnover are substantial and intentions to leave among staff may manifest as lowered performance. We examined whether team climate, as indicated by clear and shared goals, participation, task orientation and support for innovation, predicts intention to leave the job and actual turnover among hospital employees.

**Methods:**

Prospective study with baseline and follow-up surveys (2–4 years apart). The participants were 6,441 (785 men, 5,656 women) hospital employees under the age of 55 at the time of follow-up survey. Logistic regression with generalized estimating equations was used as an analysis method to include both individual and work unit level predictors in the models.

**Results:**

Among stayers with no intention to leave at baseline, lower self-reported team climate predicted higher likelihood of having intentions to leave at follow-up (odds ratio per 1 standard deviation decrease in team climate was 1.6, 95% confidence interval 1.4–1.8). Lower co-worker assessed team climate at follow-up was also association with such intentions (odds ratio 1.8, 95% confidence interval 1.4–2.4). Among all participants, the likelihood of actually quitting the job was higher for those with poor self-reported team climate at baseline. This association disappeared after adjustment for intention to leave at baseline suggesting that such intentions may explain the greater turnover rate among employees with low team climate.

**Conclusion:**

Improving team climate may reduce intentions to leave and turnover among hospital employees.

## Background

The health care sector is currently undergoing changes throughout the western world. The demographic structure of the population is changing; the number of patients in general and of patients with co-morbidities and of higher acuity is increasing [1]. Furthermore, the pressure on governments to reduce health care costs whilst improving quality continues [1-3]. As a result, the everyday work of hospital staff has become more demanding, which has lead to crises in recruitment and retention in the health care workforce. This situation is unlikely to improve in the near future because the retirement rate of the current staff will increase dramatically within the next ten years [4,5].

Intention to leave a job refers to the intent or predisposition to leave the organization where one is presently employed [6]. Although intention to leave does not necessarily mean actual employee turnover, intention has been found to be a strong predictor of quitting a job [4,7-12]. High intention to leave may also have indirect negative influences at work in the form of withdrawal, i.e. declining participation in a job [4,11]. Withdrawal has been found to manifest itself as lateness, absenteeism, avoidance behaviour, and lowered performance [4,11-14]. In hospitals, the costs of employee turnover, both direct (costs of retraining a new employee) and indirect (costs of postponing patient treatment due to lacking staff) are substantial [15]. Therefore, studying the antecedents of intention to leave among hospital employees is of high importance.

The quality of team climate may have a role in employees' intentions to leave. In previous longitudinal studies, team climate manifested as clarity of and commitment to objectives, participation, task orientation, and support for innovation have been linked to several positive outcomes at work, such as low sickness absence rates among physicians [16], and high levels of innovation in top management teams [17,18]. High levels of support for innovation experienced in teams also predicted team effectiveness over time [19]. This study on hospital employees is the first to prospectively examine the extent to which team climate is associated with intentions to leave and actual staff turnover.

## Methods

### Study population

Between 2000 and 2002, a postal questionnaire on team climate and intention to leave was sent to all of the personnel employed by 21 Finnish hospitals, a total of 3,577 men and 18,361 women. Of these, 70% responded (1,941 men, 13,405 women). Respondents who were still working in the hospitals 2 to 4 years later (1,546 men, 10,430 women) were sent a follow up questionnaire in 2004. A total of 1,134 men and 8,711 women (82%) responded to this survey.

The present study focused on two cohorts. The first cohort was set up to examine poor team climate as a predictor of actual leaving the job. Thus, we selected those 6,441 respondents (785 men, 5,656 women) who had a permanent job contract, responded to baseline survey, reported no intention to *completely *give up working at baseline, and were younger than 55 at follow-up (called the permanent employees cohort). This age range was chosen to exclude employees from the study who will retire due to age. Non-permanent employees were excluded as their job contract may have expired in spite of their willingness to stay. Second, to examine team climate as a predictor of intention to leave at follow-up, we selected those 5,098 (555 men, 4,543 women) participants who did not leave their workplace by follow-up. They are called the non-leavers. All other inclusion criteria were the same for this group as those in the permanent employees cohort except that response to follow-up survey was required. Moreover, we included both permanent and temporary employees in the non-leavers. The two cohorts were not mutually exclusive.

### Team climate

Team climate has been examined in a range of settings using the Team Climate Inventory (TCI) [20]. Studies among different occupational groups and in different countries report the validity and reliability of the tool [21-23]. As in a number of previous studies [16,24,25], we measured team climate as a one dimensional construct with a short version of TCI, the 14-item team climate inventory which has previously been validated [26,27]. The inventory taps the extent to which members of a work unit share and accept common goals, interact with each other, and develop performance together (Table 1). Responses for the items are provided on 5-point Likert-type scales (eg, 1 = strongly disagree to 5 = strongly agree). The internal consistency of the scale was highly satisfactory (Cronbach alpha reliability coefficient 0.90).

**Table 1:** Items of the Team Climate Inventory, short version.

**Item**
1. How far are you in agreement with the objectives of your work unit?
2. To what extent do you think objectives of your work unit are clearly understood by other members of the work unit?
3. To what extent do you think objectives of your work unit can actually be achieved?
4. How worthwhile do you think these objectives are to the organization?
5. We have a "we are together" attitude
6. People keep each other informed about work related issues in the work unit
7. People feel understood and accepted by each other
8. There are real attempts to share information throughout the work unit
9. Are members of your work unit prepared to question the basis of what the work unit is doing?
10. Does the work unit critically appraise potential weaknesses in what it is doing to achieve the best possible outcome?
11. Do members of the work unit build on each other's ideas to achieve the best possible outcome?
12. People in this work unit are always searching for fresh, new ways of looking at problems.
13. In this work unit we take the time needed to develop new ideas.
14. People in the work unit cooperate to help develop and apply new ideas.

Two indicators of team climate were computed based on responses: self-reported team climate (mean score of respondent's responses to the scale items) and co-worker assessed team climate. To construct the co-worker assessed team climate measure, the work unit of each respondent was identified from the employers' records. Co-worker team climate was calculated as the mean of all co-workers' team climate scores in the respondent's work unit and this mean score was then assigned to the respondent (note that respondent's own score had no effect on co-worker assessed team climate).

### Intention to leave

Intention to leave a job was measured by the following question: "What would you rather do if your livelihood were sufficient [6]?" Responses are provided on a 4-point scale that included the following choices: 1 = "I would continue working in this organization;" 2 = "I would switch to another organization in the same occupational field;" 3 = "I would switch to another occupational field," and 4 = "I would give up working completely." We used the item as a dichotomous variable: the two groups were (1) those respondents who reported that they would continue working in the same job (response option 1) and (2) those respondents who would choose to leave (options 2 and 3). Those responding that they would give up working completely (option 4) were excluded from the study (1,239 individuals from the 7,680 permanent employees and 1,782 individuals from the 6,880 non-leavers). Evidence for the validity of this measure has been previously provided [28,29], and in the present study intention to leave was a strong predictor of actual leaving the job.

### Covariates

Covariates were variables that have been correlated with intention to leave in previous studies: age [30,31], gender [4,32], organizational tenure (years employed by the organization) [4,33,34], and socioeconomic position (SES, upper non-manual, lower non-manual, manual according to the Statistics Finland Occupational Classification) [35-37]. We also measured type of employment (0 = permanent, 1 = temporary) as a covariate. To control for the effect of health on intention to leave, we assessed minor psychiatric morbidity with the 12-item version of the General Health Questionnaire (GHQ) (Cronbach alpha 0.89; cases were those who scored 4 or higher on the questionnaire) [38], and self-rated health by the question: "What is the state of your health?" (a 5-point response scale [1 = good, 2 = rather good, 3 = average, 4 = rather poor, 5 = poor] which was dichotomized and used as an indicator of poor health [options 3,4 and 5] vs not [options 1 and 2], as in previous studies) [39-44]. The 12-item GHQ has been validated against standardized psychiatric interviews [45] and self-rated health (dichotomised as in this study) has predicted mortality in various adult populations [42-44]. Comparisons with other health measures suggest that dichotomised self-rated health may be an even more inclusive and accurate measure of overall health status than medical records or self-reports of these records [43]. According to previous studies, poor health is strongly associated with considerations of leaving the job [46].

### Statistical analysis

According to the prerequisites of multilevel analyses, our dataset included individuals (employees) nested within work units. Using the multilevel analysis we were able to take this hierarchical structure of the data set into account and include both individual and work unit-level predictors in the models. The only work-unit level predictor in this study was co-worker assessed team climate. To test within-unit (interrater) agreement in assessment of team climate, that is, the extent to which raters assign the same ratings to a single target, we computed a *r*_*wg *_index [47]. This index compares the observed variance in the raters' responses to the variance that would be expected if the ratings were characterized by uniformly distributed error. A *r*_*wg *_equal to 1 would indicate that all judgements about rated subject were similar. The more there is decline in the *r*_*wg *_index close to 0, the wider there is the divergence of the opinions on the issue. When the index is 0, the suitability to use aggregated individual-level scores as indicators of group-level constructs is less obvious or unsubstantial. Therefore, a demonstration of interrater agreement further provides the measurement justification for using aggregated individual-level data as indicators of group-level constructs. An *r*_*wg *_index value of 0.7 is perceived as a limit value. In the present data, *r*_*wg *_index value for team climate was 0.96 justifying the use of co-worker assessed team climate.

Descriptive statistics included means and frequencies of study variables for both cohorts and Pearson correlations between variables among the non-leavers. Logistic regression analyses with generalized estimating equations (GEE) method with work unit as the second level [48] were performed to estimate the associations of individual assessed team climate at baseline and co-worker assessed team climate at follow-up with intention to leave at follow-up in the non-leavers cohort and the associations of intention to leave and individual assessed and co-worker assessed team climate at baseline with leaving the organization by follow-up in the permanent employee cohort. Odds ratios and their 95% confidence intervals (CIs) for team climate measures were calculated per 1 unit decrease in the score and they were adjusted for covariates. As there were no statistically significant interactions between team climate and gender on intent to leave or actual leaving the post, all analyses were based on a sample combining men and women. For these analyses we used the multilevel GENMOD GEE-estimating procedure in SAS V8 program package.

## Results

Table 2 presents characteristics of the two cohorts. Almost 90% were women and the mean age was 41 or 42 depending on the cohort. Over 80% rated their health as good and almost 80% had no psychiatric morbidity. Among the permanent employees, 37% reported intention to leave at baseline and 13% actually left their post by follow-up. Among the non-leavers, 31% reported intention to leave at baseline and 38% at follow-up.

**Table 2:** Characteristics of employees.

	All permanent respondents (N = 6441)			Non-leavers* (N = 5098)		
		
	N	%	Mean (SD)	N	%	Mean (SD)
**Baseline**						
Gender						
- Women	5656	88		4543	89	
- Men	785	12		555	11	
Mean age, yrs	6441		42.0 (6.2)	5098		40.8 (7.1)
Mean tenure, yrs	6361		14.2 (7.2)	5009		12.7 (7.8)
Socioeconomic status						
- Upper non-manual	1027	16		764	15	
- Lower non-manual	4636	72		3793	74	
- Manual	778	12		541	11	
Type of employment contract						
- Permanent	6441	100		3852	76	
- Temporary				1241	24	
Self-rated health						
- Good	5324	83		4363	86	
- Average or worse	1075	17		700	14	
Minor psychiatric morbidity						
- Non-case	4984	78		4025	79	
- GHQ-case	1433	22		1055	21	
Team climate, self reported	6441		3.4 (0.6)	5098		3.5 (0.6)
Team climate, co-worker assessed	6441		3.5 (0.3)			
Intention to leave						
- No	4062	63		3518	69	
- Yes	2379	37		1580	31	
Left the hospital by follow-up						
- No	5598	87		5598	100	
- Yes	843	13		0	0	
**Follow-up**						
Left the hospital by follow-up						
- No	5598	87		5598	100	
- Yes	843	13		0	0	
Team climate, co-worker assessed	N/A			5098		3.5 (0.3)
Intention to leave	N/A					
- No				3161	62	
- Yes				1937	38	

*Employees who worked in the target hospitals at baseline and follow-up including temporary employees.

N/A, not applicable.

Table 3 shows zero-order correlations between study variables among the non-leavers. Higher individual assessed team climate at baseline was associated with better self-rated health at baseline, higher co-worker assessed team climate at follow-up and lower intention to leave at baseline and follow-up. There was a significant but weak cross-sectional association between higher co-worker assessed team climate and lower intention to leave at follow-up.

**Table 3:** Pearson correlation coefficients between study variables among non-leavers (n = 5098).

	1	2	3	4	5	6	7	8	9	10
**Baseline**										
1 Gender										
2 Mean age	-.03									
3 Mean tenure	-.09***	.62***								
4 Socioeconomic status	-.11***	-.08***	.02							
5 Type of employment contract	.02	-.42***	-.49***	-.02						
6 Self-rated health	.00	.14***	.12***	.06***	-.08***					
7 Minor psychiatric morbidity	-.01	.05*	.05**	-.00	-.05**	.20***				
8 Team climate, self reported	-.00	-.04*	-.02	-.08***	.04*	-.11***	-.19***			
9 Intention to leave	.01	-.06***	-.01	-.00	-.04	.09***	.18***	-.26***		
**Follow-up**										
10 Team climate, coworker assessment	-.00	-.02	-.03	-.01	.01	-.03	-.04*	.22***	-.05**	
11 Intention to leave	-.01	-.07***	-.04*	-.00	-.03	.05***	.14***	-.19***	.39***	-.08***

* p < .01

** p < .001

*** p < .0001

Table 4 shows the association between team climate and intention to leave the organization at follow-up among the non-leavers. For those who had no intention to leave at baseline, lower self-reported team climate predicted higher likelihood of having such an intention at follow-up (odds ratio per 1 standard deviation decrease in team climate was 1.6). Lower co-worker assessed team climate at follow-up was also strongly association with higher intention to leave (odds ratio 1.8). Both these associations remained in a model including all baseline characteristics and both team climate measures. Among those who already had the intention to leave the organization at baseline, the associations between team climate and intention to leave at follow-up were in similar direction but weaker.

**Table 4:** Team climate as a predictor of intention to leave at follow-up by intention to leave at baseline among non-leavers.

	**Odds ratio (95% CI) for intent to leave at follow-up, adjusted for**		
	
	Gender, age, SES (A)	A+ tenure, type of employment, self-rated health, GHQ (B)	B + self/co-worker assessed team climate
**No intention to leave at baseline (n = 3402 to 3518)**			
Team climate, self reported*	1.60 (1.40 to 1.84)	1.54 (1.34 to 1.77)	1.47 (1.27 to 1.70)
Team climate, co-worker assessment*	1.80 (1.36 to 2.39)	1.79 (1.35 to 2.38)	1.52 (1.13 to 2.05)
**Intention to leave at baseline (n = 1551 to 1580)**			
Team climate, self reported*	1.23 (1.04 to 1.45)	1.15 (0.97 to 1.37)	1.12 (0.94 to 1.33)
Team climate, co-worker assessment*	1.48 (1.01 to 2.18)	1.46 (0.98 to 2.18)	1.40 (0.93 to 2.10)

*Per 1 unit decrease in the scale (range from 1 to 5). Self-reported team climate is based on baseline data and co-worker assessed team climate is based on follow-up data.

Results on predictors of actual leaving the job are presented in Table 5. Among employees with a permanent job contract at baseline, the odds for leaving the job by follow-up were 1.8 times higher for those with an intention to leave at baseline compared to those with no such an intention. Adjustment for other baseline characteristics had little effect on this association. The likelihood of leaving the organization by follow-up was also greater for those employees who reported low team climate at baseline. This association disappeared after adjustment for intention to leave at baseline suggesting that such intentions may in part explain the greater turnover rate among employees with low self-reported team climate. Odds for leaving was also elevated among employees with low co-worker assessed team climate at baseline, but this association did not reach statistical significance.

**Table 5:** Intention to leave and team climate at baseline as predictors of leaving the job by follow-up among employees with a permanent job contract at baseline (n = 6299 to 6441).

**Baseline characteristics**	**Odds ratio (95% CI) for turnover by follow-up, adjusted for**		
	
	Gender, age, SES (A)	A+ tenure, self-rated health, GHQ (B)	B + team climate/intention to leave†
Intention to leave			
- No	1.00	1.00	1.00
- Yes	1.82 (1.57 to 2.12)	1.87 (1.59 to 2.19)	1.82 (1.54 to 2.14)
Team climate, self reported	1.23 (1.08 to 1.40)	1.22 (1.07 to 1.40)	1.09 (0.94 to 1.25)
Team climate, co-worker assessment.	1.20 (0.86 to 1.68)	1.22 (0.87 to 1.71)	1.17 (0.83 to 1.63)

*Per 1 unit decrease in the scale (range from 1 to 5). Self-reported and co-worker assessed team climate scores are based on baseline data.

†Odds ratio for intention to leave is adjusted as in model B and additionally for self-reported team climate and co-worker assessed team climate. Odds ratios for self-reported team climate and co-worker assessed team climate are adjusted as in model B and additionally for intention to leave.

No strong evidence was found that the associations of team climate with intention to leave and actual leaving the job would be attributable to some specific sub-component of team climate. We repeated the analysis in Table 4 by replacing self-reported team climate score with four subscale scores. The odds ratios for all four subscales were smaller than that for the total scale, i.e., 1.06 (95% CI 0.89 to 1.25) for vision, 1.14 (95% CI 0.99 to 1.32) for participatory safety, 1.07 (95% CI 0.91 to 1.25) for task orientation, and 1.18 (95% CI 1.04 to 1.34) for support for innovation compared with 1.80 (95% CI 1.36 to 2.39) for total team climate. This was also the case for actual leaving the job (analyses in Table 5), with the corresponding odds ratios being 1.05 (95% CI 0.91 to 1.21) for vision, 1.12 (95% CI 0.99 to 1.27) for participatory safety, 1.00 (95% CI 0.87 to 1.16) for task orientation, and 1.03 (95% CI 0.92 to 1.16) for support for innovation versus 1.23 (95% CI 1.08 to 1.40) for total team climate.

## Discussion

In this large contemporary sample of hospital employees, poor team climate was associated with intention to leave the current post. This association was seen both for self-reported and co-worker assessed team climate and it was not accounted for by other factors, such as age, gender, occupation, tenure, health and psychiatric morbidity.

It has been hypothesized that intention to quit is a proximal precursor of turnover and that work-related factors may represent more distal causes in the withdrawal process [12]. Our findings are consistent with this general hypothesis. First, intention to leave strongly predicted actual leaving the job, a finding also reported in a number of other studies [4,7-9]. Second, poor self-reported team climate predicted leaving the job, but this association attenuated towards the null after adjustment for intention to leave. Such an effect attenuation supports the possibility that intention to leave in part mediates the association between poor team climate and leaving the job [49]. Third, the effect of team climate on intention to leave at follow-up was stronger among those who initially did not have such an intention than among those who had. This demonstrates that team climate indeed precedes a change in intentions to leave.

Although this is probably the first study on team climate, intention to leave and turnover, there are several previous studies reporting associations of organizational climate and work group cohesion with withdrawal thoughts and behaviours [4,50-52]. Organizational climate and work group cohesion are conceptually close to team climate, but they might provide a less practical basis for planning interventions to reduce turnover [17]. In general, our findings extend the evidence on psychosocial factors that have a significant role in people's decisions to continue working in their current workplaces. Previous studies have found that stressful aspects of work, such as high levels of job tension [52], stress [30], role conflict [6,32,53], role ambiguity [54,55], role insufficiency [6], emotional exhaustion [54], workplace bullying [56], and low job control [57] are related to increased intention to leave. Career moves, rewards, and performance levels are also suggested to play a role in employee decisions about staying or leaving [57-60]. In addition, empowerment, supervisor behaviour and relationships with supervisors and managers have been shown to contribute to employee retention [14,34,36,61,62].

In addition to self reports, we used co-worker assessment of team climate, a measure based on aggregated work unit data. A high perceptual agreement provided a justification for the use of this group-level indicator in multilevel analysis that took into account the hierarchical structure of these data. The finding that both self-reported and co-worker assessed team climate were associated with intention to leave is important from conceptual, methodological, and practical point of views. First, the result demonstrates that the effects of team climate reflect influences related to shared perceptions of organizational members about the work environment – not only the impacts of individual's subjective perceptions. Second, we were able to reduce bias arising from differences in response styles, because co-worker assessed team climate is an inferred measure independent of participant's own perceptions. The possibility that observed associations were inflated by common-method variance problems was also reduced. Third, the independent effects of self-reported and co-worker-assessed team climate imply that interventions at individual and group levels might be useful in improving team climate and reducing intentions to leave.

A recent literature review suggests that much of the turnover research is characterized by small samples sizes and cross-sectional data [4]. Longitudinal study design with a large cohort is a strength of this study because it enabled robust determination of the temporal order between team climate, intent to leave and actual leaving the job. Such data largely eliminate reverse causality (i.e., intention to leave affecting perceptions of team climate) as an explanation for observed associations. The response rates for the two surveys were 70% and 82%, respectively, which are satisfactory for studies of this kind [63].

Our sample was predominantly female corresponding to the gender distribution among hospital personnel in Finland and elsewhere. This study was conducted in one country, and it is therefore tied to the Finnish context. Other drawbacks of our study include homogenous sample (almost exclusively white Finns) and the restricted follow-up period (i.e., 2 to 4 years). Thus, further research is needed to confirm our results before they can be generalised to other countries, other contexts and multiple ethnic groups. A longer follow-up period would help in determining how distant a determinant of turnover team climate is. This may have practical relevance, as identifying an early determinant provides time to intervene and potentially reduce the rate of turnover.

## Conclusion

Governments are facing an increasing shortage of skilled health care staff in many western countries. Therefore, the ability of health care to retain its workforce is particularly important. Our evidence suggests that development of team climate should be considered as a potential target in interventions to reduce staff turnover.

## Competing interests

The author(s) declare that they have no competing interests.

## Authors' contributions

MK, AV, JP, HL and JV developed the study design, JP performed statistical analyses, and MK and AV drafted and all authors edited the manuscript. MV and ME participated in the design and MK, MV and JV participated in the administration of the original surveys. MK is the principal investigator of the Hospital Personnel study. All authors read and approved the final manuscript.

## Pre-publication history

The pre-publication history for this paper can be accessed here:


